# Long term inguinal pain comparing TEP to Lichtenstein repair: the TEPLICH RCT 8 years follow-up

**DOI:** 10.1007/s10029-024-03246-y

**Published:** 2024-12-19

**Authors:** A. Gutlic, U. Petersson, P. Rogmark, A. Montgomery

**Affiliations:** 1 Deparment of Urology, Skåne University Hospital Malmö, University of Lund, Malmö, Sweden; 2 Department of Surgery, Skåne University Hospital Malmö, University of Lund, Malmö, Sweden; 3 University of Lund, Lund, Sweden

**Keywords:** Inguinal hernia, TEP, Lichtenstein, Chronic pain, QoL, Recurrence

## Abstract

**Purpose:**

To investigate long-term chronic postoperative inguinal pain (CPIP), QoL and recurrence in patients with a primary inguinal hernia comparing TEP to Lichtenstein.

**Material and methods:**

A questionnaire-based follow-up containing the Inguinal Pain Questionnaire (IPQ), the Cunningham Pain Scale and SF-36 was done 8 years after the TEPLICH RCT. The main objective was non-ignorable pain last week according to IPQ. A phone interview was performed with patients reporting new non-ignorable pain and those having a suspected recurrence. Records were scanned for long-term CPIP and recurrences. A lost to follow-up analysis was performed.

**Results:**

A total of 322 of 366 patients (88%) completed the follow-up of mean 7.94 years (5–10.75 years). Non-ignorable pain last week was reported by 7.6% in TEP and 6.7% in Lichtenstein (p < 0.73). New non-ignorable pain was reported by 5 patients. No difference in non-ignorable pain over time (1–8 years) was observed within groups. Moderate to severe pain, according to Cunningham, was reported by 3.8% in TEP and 5.5% in Lichtenstein (p < 0.48). QoL remained above the Swedish norm. No recurrences occurred after 3 years follow-up. The lost to follow-up analysis showed no difference in non-ignorable pain.

**Conclusions:**

RCTs, comparing TEP to Lichtenstein repair with follow-up ≥ 5 years regarding CPIP are sparse with conflicting data. In this study, low frequencies of CPIP present at 3 years seem to persist at 8 years. Recurrences occured within the first 3 years. Patients need to be informed of the risk of long-term CPIP.

## Introduction

Chronic Postoperative Inguinal Pain (CPIP) has been defined as pain persisting for more than 3 months after surgery for inguinal hernia [1]. The incidence is estimated to be 10–12% [2] but even higher incidences are described. A recent meta-analysis by Aiolfi et al. reported chronic pain rates of 3.1% for TEP and 9.4% for Lichtenstein, based on data from 20 RCTs [3]. Most patients with CPIP experience mild pain and only a few percent have more severe pain [4, 5]. Nevertheless, when present it is a main adverse outcome that may persist for many years after any inguinal hernia repair and represents a greater problem than recurrences today since there is no treatment that can alleviate CPIP with certainty unless the cause is a recurrent hernia.

Most studies on sequelae after TEP and Lichtenstein repair report CPIP and recurrence 1–3 years after surgery. The randomized controlled TEPLICH trial reported no difference in pain or recurrence rates between TEP and Lichtenstein 1 and 3 years postoperatively [6]. A meta-analysis including 21 RCTs on TEP vs Lichtenstein repair confirm equal result regarding chronic pain between techniques but report a higher recurrence rate for TEP than for Lichtenstein, contrary to the TEPLICH 3-year results [7]. Notably, one RCT included in this meta-analysis used a smaller mesh size for TEP than recommended, once this study was excluded, no difference in recurrences between techniques were observed [8, 9]. Four of the studies in the meta-analysis report 5 years follow-up [10–13], and only 1 study report a follow-up exceeding 7 years [14]. In these 5 studies, the incidences of CPIP after TEP range from 0 to 28.0% and after Lichtenstein from 6.8 to 14.9%. The corresponding incidences for recurrences are 0–5.1% and 1.2–8.1%, respectively. Frequency and magnitude of pain are inconsistent between studies due to different instruments used for pain assessment [15]. Hence, there is still an uncertainty whether CPIP may mitigate, disappear, or develop more than 5 years after surgery.

CPIP, as well as the incidence of recurrence, may be dependent on the operative experience of the surgeon [2, 16, 17], and standardized technical performance is of importance to minimize the risk. Several studies in the above-mentioned meta-analyses, including the TEPLICH trial, reported having department-certified surgeons following standardized protocols when performing either technique but no study except the TEPLICH trial report on CPIP over time in the individual patient [10, 11, 14, 18–21].

The objectives of this study were to investigate long-term chronic pain, recurrence, and quality of life (QoL) comparing TEP to Lichtenstein in patients included in the TEPLICH randomized controlled trial, and to analyze pain pattern over time for the individual patient to describe possible long-term harm of surgery. In the TEPLICH trial department-certified hernia surgeons trained according to the same curriculum performed the operations.

## Methods

A long-term questionnaire-based follow-up of patients in the TEPLICH trial was initiated in February 2019. The Inguinal Pain Questionnaire (IPQ), the Cunningham pain scale, the Short Form 36 (SF-36), and study specific questions were sent to eligible patients by mail. Eligible were patients previously included in the randomized controlled TEPLICH trial (men 30–75 years, ASA I–II, with a primary unilateral inguinal hernia) and not lost to follow up due to declining further participation, recurrence, death, moving abroad, and paralysis up to three years postoperatively [6]. Patients in the TEPLICH trial had previously answered the same questionnaires preoperatively, and at 1 and 3 years postoperatively. One reminder was sent to those that did not reply. Technical details regarding the procedures are described in an earlier report from the TEPLICH trial [6].

A chart review was performed for all patients and scrutinized for CPIP and recurrence by checking all available in- and out-patient hospital records. Patients participating in the study were informed to contact the surgery out-patient clinic for any symptoms related to the groin, thus primary care records were not searched.

The objective was to compare *non-ignorable pain last week* according to the IPQ between the TEP and Lichtenstein groups. Other objectives were to compare *non-ignorable pain right now* (IPQ), *any pain last week* and *right now* (IPQ), pain according to the Cunningham pain scale, and SF-36 physical and mental composite scores between groups. Furthermore, each individual patient’s pain status over time was assessed as *non-ignorable pain last week* from before the operation to the present follow-up.

### Pain instruments

Pain was assessed using the IPQ [22] and the Cunningham pain scale [23]. The IPQ question regarding *pain right now* and the question regarding *pain last week*, with seven grades of increasing pain, were used to identify patients with pain. The cut-off for any pain was set to grade 2 (*pain present but can easily be ignored*) or higher and the cut-off for non-ignorable pain to grade 3 (*pain present, cannot be ignored but does not interfere with everyday activities*) or higher, and were chosen in accordance with the previous TEPLICH report [6]. A study specific question regarding whether the patients had developed any discomfort in the operated groin that they did not have prior to surgery, was used to capture other problems than pain. The cut-off for pain in the Cunningham pain scale was chosen as moderate pain (*pain that did not allow return to all normal preoperative activities*) or worse.

### New non-ignorable pain and new discomfort

The patients´ previous questionnaire answers, i.e. preoperative, and at 1 and 3 years postoperative, were scrutinized to assess new pain or new discomfort.

### SF-36

The Short Form 36 (SF-36^®^; Quality Metric, Lincoln, Rhode Island, USA), licensed for the overall TEPLICH study by the HRQoL group at Gothenburg University, was used to measure Health-Related QoL. The composite scores for physical and mental health were calculated according to the SF-36^®^ manual.

### Telephone interview and clinical examination

According to the TEPLICH trial protocol, clinical examinations had been carried out at the 1- and 3 years follow-up. In this long-term follow-up, patients reporting new *non-ignorable pain last week* or *new discomfort*, and patients with a suspected recurrence found during chart review were planned for a telephone interview. The purpose of the interview was to further elucidate the degree of pain or discomfort and symptoms indicating recurrence as well as time of occurrence. Questions asked at the telephone interview were: *have you experienced discomfort or new/increasing pain in the operated groin? Have you noticed a bulge or lump in the operated groin at rest or when sneezing/coughing? When did the symptoms occur?* Multiple attempts were done to contact the patients. When failing to contact the patients, the available questionnaire answers were used for statistical calculations. In case of discrepancies between patient´s answer in the questionnaire and the telephone interview, the latter information being the latest and most detailed, was used for the statistical calculations. Patients reporting new pain, new discomfort or symptoms indicating a recurrence during the telephone interview were thereafter planned for a clinical examination at the out-patient clinic.

### Lost to follow-up analysis

An analysis was conducted where patients lost to follow-up between 1 year and the present follow-up were compared to patients remaining for the present follow-up. Differences between these groups at 1 year in non-ignorable pain last week and age were compared for both techniques.

### Sample size calculation

At the start of the TEPLICH study a sample size calculation was carried out assuming that 20% of the Lichtenstein patients and 8% of the TEP patients would have *non-ignorable pain last week* according to IPQ one year after surgery, which constituted the study’s primary endpoint. At that time a power calculation for an 8 year follow-up was not performed. When calculating the sample size retrospectively with the same presumed frequencies of *non-ignorable pain last week*, a significance level of 0.05 and a power of 80%, 131 patients would be needed in each arm.

### Statistical analysis

Statistical analyses were performed using IBM SPSS 25. Mean and standard deviation (SD) or median and interquartile range (IQR) values were used as appropriate. Pearson chi square or Fischer exact test (2-sided) and McNemar tests were used when comparing categorical and binary data. Student *t* test was used for continuous data. A p value of 0.05 was deemed significant. SF-36 norm-based scores were calculated using the Swedish age- and sex-specific mean and SD. The normalized data have a mean (SD) value of 50 [10]. A 5-point difference corresponds to an effect size (Cohen’s *d*) of 0.5, which is regarded as a medium-sized clinical difference.

## Results

**Fig. 1:**
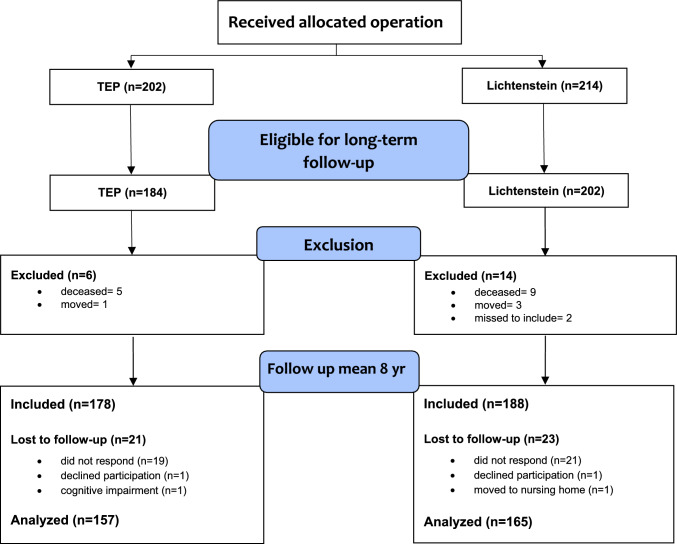
Flowchart

Of the 416 patients receiving the allocated operation 322 (77%), 157 in the TEP and 165 in the Lichtenstein group, remained for analysis at a mean follow-up of 7.94 years (SD 1.64; range 5–10.75 years). A retrospective power calculation revealed a power of 87.6% based on the assumptions presented above. A study flow-chart with reasons for lost to follow-up is presented in Fig. 1.

### IPQ, Cunningham and SF-36^®^

**Fig. 2:**
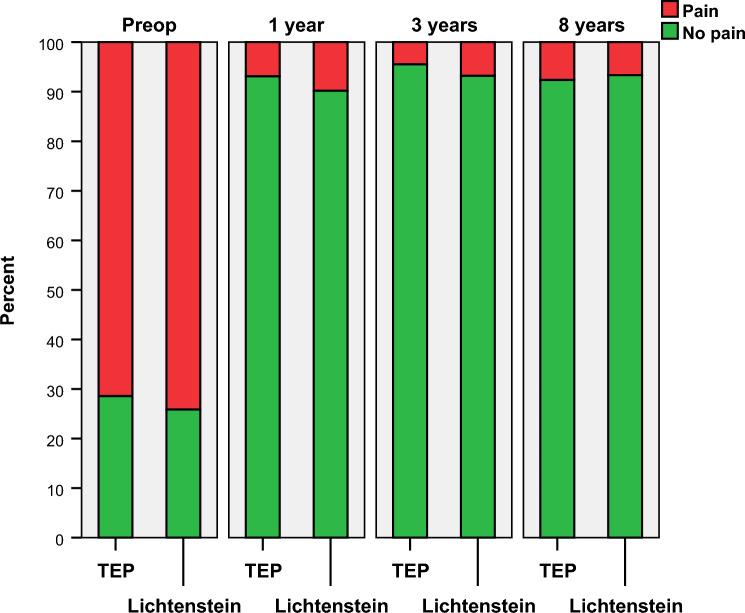
Non-ignorable pain over time comparing TEP to Lichtenstein

The objective, IPQ-reported *non-ignorable pain last week*, was reported by 12 (7.6%) in the TEP and 11 (6.7%) in the Lichtenstein group (p < 0.73). No differences in *non-ignorable pain last week* compared to 1 year was observed within groups (TEP p = 0.61 and Lichtenstein p < 0.73). Figure 2 shows the distribution of non-ignorable pain from preoperative to the present follow-up. Non-ignorable pain right now was reported by 8 (5.1%) in the TEP and 8 (4.8%) in the Lichtenstein group (p < 0.56). Any pain last week was reported by 24 (15.3%) in the TEP and 30 (18.2%) in the Lichtenstein group (p = 0.49). The corresponding figures for any pain right now were 16 (10.2%) for TEP and 20 (12.1%) for Lichtenstein (p < 0.58).

**Fig. 3:**
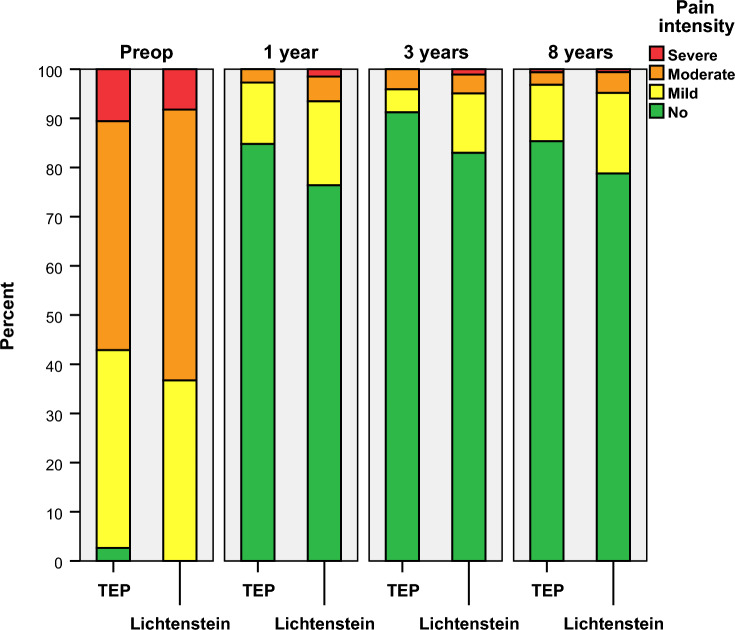
Pain over time according to Cunningham comparing TEP to Lichtenstein

Moderate or severe pain according to the Cunningham pain scale was reported by 6 (3.8%) patients in the TEP and 9 (5.5%) in the Lichtenstein group (p < 0.48) at 8 years. No differences in pain status compared to 1 year was observed within groups (TEP p < 0.73 and Lichtenstein p < 1.00). Mild pain or worse was reported by 23 (14.6%) patients in the TEP and 35 (21.2%) in the Lichtenstein group (p < 0.13). Figure 3 shows the distribution of pain according to the Cunningham pain scale from preoperative to the present follow-up.

**Fig. 4:**
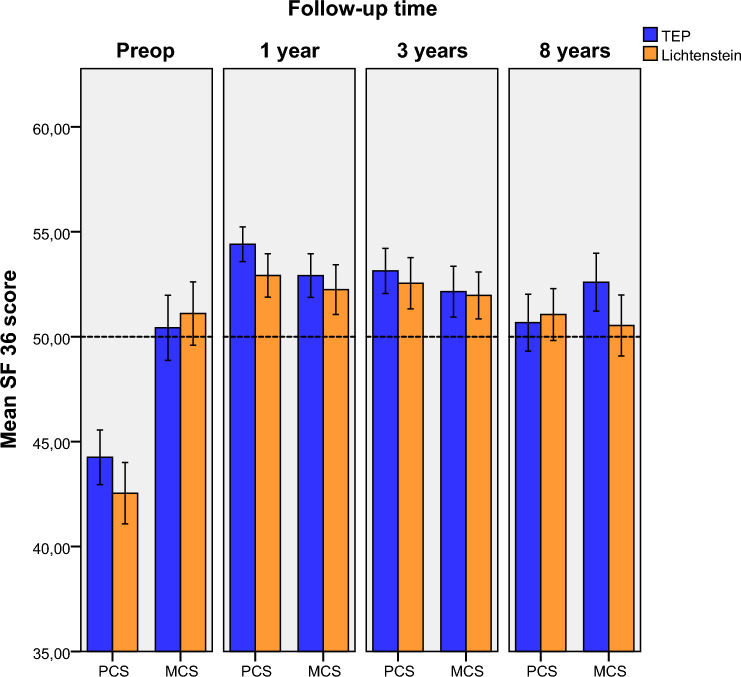
The distribution of PCS and MCS from preoperative to 8 years

All patients continued to perform above the Swedish norm in the SF-36 physical and mental composite scores after surgery. The physical composite score (PCS) decreased between 1 year and the present follow-up in both groups (TEP p < 0.001 and Lichtenstein p < 0.001). The mental composite score (MCS) was unchanged for TEP but decreased for Lichtenstein between 1 year and the present follow-up (TEP p < 0.67 and Lichtenstein p < 0.003). Figure 4 shows the distribution of PCS and MCS from preoperative to the present follow-up.

### New non-ignorable pain and new discomfort

A total of 6 patients reported new non-ignorable pain last week and 12 patients reported new discomfort in the questionnaire. Five patients with new pain last week reported it as grade 3 (“pain present but does not interfere with everyday activities”) and one as grade 5 (“pain present that interferes with most activities”). When interviewed by telephone, 1 patient denied having pain or signs of recurrence, despite reporting pain in the questionnaire. The remaining 5 patients were not possible to reach. Out of the 12 patients reporting new discomfort, 5 patients denied having discomfort, pain or sign of a recurrence when interviewed. The remaining 7 patients were not possible to reach.

In the additional chart review no hospitalization or visits at the out-patient clinic related to groin pain were noted. No reoperations were performed.

### Pain over time and surgical harm

**Fig. 5:**
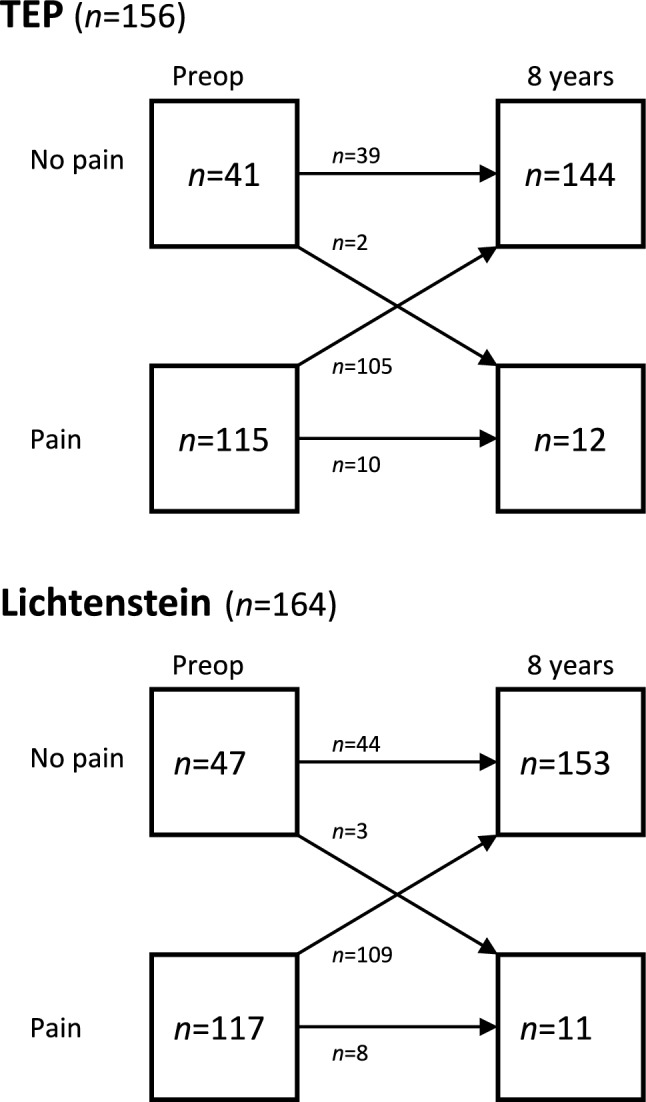
Individual patients’ pain status (non-ignorable pain) from preoperative to 8 years

For patients available for long-term follow-up, *non-ignorable pain last week* changed from 73.2% in TEP and 71.0% in Lichtenstein preoperatively to 5.7% and 9.7% at 1 year, 3.8% and 6.1% at 3 years and 7.6% and 6.7% at 8 years, respectively. No differences between groups regarding *non-ignorable pain last week* were seen throughout the study. Of the patients remaining for the present follow-up and did not have pain preoperatively, 2 (4.9%) patients in TEP and 3 (6.4%) patients in Lichtenstein reported pain after 8 years. The pain arose between 3 and 8 years in all patients. The corresponding number of patients that was relieved from their preoperative pain was 91.3% and 93.2%, respectively. Patients with preoperative pain reported persisting pain in 8.7% and 6.8%, respectively. Figure 5 summarizes pain disappearance and development from preoperative to the present follow-up.

### Cumulative recurrences

During the entire study a total of 6 patients, 4/157 (2.5%) in TEP and 2/165 (1.2%) in Lichtenstein, were diagnosed with a recurrence, 5 at 1 year and 1 at 3 years follow-up with no difference between groups (p < 0.36). No further recurrences were noted between 3 years and the present follow-up.

### Lost to follow-up analysis

A total of 72 of 392 patients (18.4%) were lost to follow-up between 1 year and the present follow-up.

Four of 31 TEP patients (12.9%) lost to follow-up reported *non-ignorable pain last week* at the 1 year follow-up, compared to 9 of 157 patients (5.7%) remaining for the present follow-up (p < 0.234). Corresponding figures for Lichtenstein were 4 of 41 (9.8%) lost to follow-up and 16 of 163 (9.8%) of those remaining for the present follow-up (p < 1.00). No significant age difference were seen between those lost to follow-up and those remaining.

## Discussion

This is a long-term, mean 8 years, follow-up of a randomized controlled trial comparing TEP to Lichtenstein concerning chronic pain, QoL and cumulative recurrence. Existing long-term reports from RCTs and prospective trials reporting on chronic pain 5 years or longer after inguinal hernia surgery are inconclusive regarding whether pain persists or fades [4, 11, 12, 24–36]. The instruments used to measure pain differ between these studies and some of them fail to report a clear cut-off for pain. Further limitations in some studies are small cohorts and large drop-out rates. We present a long-term follow-up of the TEPLICH RCT with clear definitions of pain, described with two different pain scales, and an appropriate cohort size with a drop-out rate of 23% after 8 years.

The objective for this report was to compare *non-ignorable pain last week* according to the IPQ questionnaire as in the previous TEPLICH report [6]. We chose to focus on *pain last week* to incorporate a time-period for the patients to evaluate their pain experience, as opposed to reporting *pain right now*. We also chose a cut-off level where the effect of pain was considered and not negligible, i.e. the pain should be non-ignorable. No RCT comparing TEP to Lichtenstein using IPQ as the primary modality for reporting pain 5 year or longer exist beside this.

For the patients available for this long-term follow-up, *non-ignorable pain last week* changed from 73% preoperatively to 1.6% after 8 years, and 92.2% of patients with preoperative pain was relieved from their pain, with no differences between groups throughout the study, indicating good pain relief with either technique. The benefit of repair with either of the techniques is also reflected in the increase in QoL measured as SF-36 PCS from below the norm preoperatively to above it at 8 years. The MCS was slightly above the norm both preoperatively and during follow-up. Of 87 patients without pain preoperatively, five (5.7%) reported pain at long-term follow-up. Those patients were possibly harmed by the surgical procedure with new pain appearing between 3 and 8 years. Although it is difficult to find a plausible pathogenetic explanation for this late appearing pain, those patients were possibly harmed by the surgical procedure. Since these patients could not be contacted by phone or clinically examined, other causes for late-onset pain localized in the groin area cannot be ruled out. Nevertheless, it is important to be aware of the possibility of late-onset pain and to inform the patients about the risk.

The Cunningham pain scale was the main pain instrument used by Eklund et al. in the SMIL II-study which included 1370 patients [4]. Moderate or severe pain was reported by 2.7% in TEP and 7.1% in Lichtenstein at 1 year and by 1.9% in TEP and 3.5% in Lichtenstein at 5 years in that study. Comparable figures were found in this study with 2.7% for TEP and 6.5% for Lichtenstein at 1 year and 3.8% for TEP and 5.5% for Lichtenstein at 8 years. When also including mild pain the SMIL II study reported pain in 11.0% in TEP and 21.7% in Lichtenstein at 1 year and 9,4% in TEP and 18.8% in Lichtenstein at 5 years. Our findings were similar with 15.2% in TEP and 23.6% in Lichtenstein at 1 year and 14.6% for TEP and 21.2% for Lichtenstein at 8 years. Grant et al. used a 5-grade pain scale (none, very mild, mild, severe and very severe pain) resembling the Cunningham scale and reported very mild or worse pain in 27.7% and 35.6% at 1 year for patients operated by TEP or open tension-free mesh repair, respectively [24]. At 5 years the corresponding figures were 18.1% and 20.1%. Mitura et al. compared Lichtenstein to the Valenti technique and reported a similar incidence of mild or worse pain for Lichtenstein of 26.3% after mean 9 years of follow-up in their randomized controlled trial [33].

As indicated by this and other studies the percentage of patients reporting pain mainly seem to depend on pain definition and follow-up time. Generally, pain appears to diminish over time but the frequency of any pain in studies with long-term follow-up seems to approximately be between 10 and 25% after 5 years or longer. For non-ignorable pain or pain with consequences on everyday activities the frequencies drop to below 8%. Interestingly, an RCT from the Netherlands comparing Lichtenstein to TIPP repair show otherwise [27]. Pain was measured with the visual analog scale (VAS) and defined as continuous chronic pain. Mild, moderate and severe pain was categorized as VAS 1–3, 4–6 and 7–10, respectively. 18 patients in the Lichtenstein group experienced moderate continuous pain and 2 reported severe pain at 1 year follow up but at median 7 years only 1 patient still experienced pain. A systematic review analyzing articles on CPIP found VAS to be the predominant scale used in 73% of articles [15]. It can be argued that studies that solely rely on the VAS scale as their instrument for measuring CPIP may contribute to the risk of estimating pain incorrectly due to its continuous characteristics without cut offs for reference and its lack of CPIP sensitive questions. This needs to be accounted for when drawing conclusions and comparing results between studies.

The cumulative recurrence rate in this study was 1.9% without difference between techniques and all occurred within 3 years. Berndsen et al. prospectively followed 452 patients for 5 years where 56% were operated by TEP and 12% by Lichtenstein repair. They reported a cumulative recurrence rate of 2.3% for primary hernias, which is in accordance with our results [37]. Eklund et al. reported a cumulative recurrence rate of 3.5% after TEP and 1.2% after Lichtenstein and the majority occurred within the first 3 years of follow up [10]. In a RCT by Sevinc et al. 5 recurrences (3.4%) were found in TEP and 8 (8.2%) in Lichtenstein with all occurring within the first 28 months [38]. Four additional RCTs with a follow up time of 5 years reported an overall low incidence of recurrences (1–1.9%) after Lichtenstein repair where most had occurred within 2 years and all at 3 years, except for Pierides et al. where 1 recurrence occurred at 5 years [12, 28, 35, 39]. As indicated by most of these studies with follow-up of 5 years or more the cumulative recurrence rates after both TEP and Lichtenstein are low and could be expected to be below 3% and to occur within 3 years.

This long-term follow-up study has its limitations. A lost-to-follow-up of 23% is one of those limitations. Fewtrell et al. recommended that the percentage of drop-outs in each group should be reported, the capacity of the follow-up study to identify the hypothesized outcome effect given the achieved sample should be described, differences in baseline data or measured outcomes between drop-outs and patients completing follow-up should be assessed [40]. To address these issues, a lost-to-follow-up analysis and a post-hoc power analysis were performed. We found no difference in non-ignorable pain at 1 year follow-up between drop-out patients and those remaining for long-term analyses, indicating no or little impact on the results due to drop-out in this study. Apart from our study, only Mitura et al., and Droeser et al., included a drop-out analysis but they did not include chronic pain in the analysis. In addition, with the achieved number of patients in the long-term follow-up and assuming the same 12% difference in pain between the groups as for the primary outcome in the whole TEPLICH trial [6], the post-hoc power analysis showed a power of 87.6%. Furthermore, a similar number of patients were lost to follow-up in both groups. Further, the main objective, non-ignorable pain last week at 8 years was observed in 7.6% in TEP and 6.7% in Lichtenstein (p < 0.73). With a difference of only 0.9%, a large discrepancy in non-ignorable pain would be required to reject the null hypothesis confirmed in this study. No other study comparing TEP to Lichtenstein has an 8 year follow-up. Other RCTs with a follow-up of 5 years or more report drop-our rates of 60.1%, 75.3% and 84.3%, respectively [4, 11, 24]. One small-sized RCT, including only 168 patients with a follow-up time of more than 6 years, reported a follow-up of 87.5%[14]. This highlights the challenge of achieving low drop-out rates when follow-up time exceed 5 years.

Not examining all patients clinically might be considered a weakness in the study design. A study by Vos et al. found that recurrences were not diagnosed in 54% of patients when using a questionnaire compared to a clinical examination [41]. Our patients were clinically examined at 1 and 3 years, and in this long-term follow-up only if a recurrence was suspected on chart review or during telephone interview. However, the PINQ-PHONE study indicate that a telephone interview is as sensitive as a clinical examination [42]. They followed 300 laparoscopically operated inguinal hernia patients and compared a telephone interview with a clinical examination in detecting a symptomatic or asymptomatic recurrence. A recurrence was diagnosed in a total of 5 patients using the PINQ-PHONE questionnaire and all of them were confirmed with a physical exam, hence overall sensitivity was 1.00. Another 43 patients who scored positive for a recurrence using the PINQ-PHONE questionnaire were cleared as having no recurrence after the physical exam, giving the questionnaire a specificity of 0.85 and concluding that it is reasonable to offer a physical exam to all patients having a suspected recurrence according to the questionnaire. Our telephone interview resembled the PINQ-PHONE questionnaire, except for not containing a telephone-guided Valsalva maneuver, and was offered to all patients reporting new *non-ignorable pain last week* or *new discomfort*, and patients with a suspected recurrence found during chart review. Examining all patients after such a long time is costly, time consuming, and a higher drop-out rate could be expected due to decreased patient motivation for long-term clinical follow-up. Not being able to get in touch with all the patients reporting new pain or new discomfort for a telephone interview is another mentionable weakness of the study. Another interesting point about new pain and discomfort is that some studies have shown discrepancies in pain reporting between questionnaires and phone interviews, where patients interviewed by phone reported less pain [43]. However, other studies found no difference [43]. Given this conflicting evidence, it remains unclear which method results in less collection bias. Our rationale for relying on the phone interview instead of the questionnaire when discrepancies in pain reporting between the two occurred, is based on the fact that new pain such a long time after surgery is less frequent after 5 years of follow up [44]. Even though we cannot definitively determine the cause of the late-onset pain we observed, a possible explanation could be patients’ response bias while filling in the questionnaires or an undiagnosed recurrence. This was the reason we conducted interviews with patients experiencing new pain or discomfort.

The RCT design, the use of two pain scales at all follow-up occasions creating possibilities for comparison within the study as well as with other studies are strengths of this study.

When summarizing the results of this 8 years follow-up of the TEPLICH RCT we conclude that *non-ignorable preoperative pain* was relieved in 9 of 10 patients, was still present in 7.8%, and 5.7% of the preoperatively pain free patients suffered late appearing pain. QoL was improved by surgery and normalized postoperatively. Cumulative recurrence rate was 1.9% and occurred within 3 years postoperatively. These findings did not differ between the TEP and the Lichtenstein techniques.

## Data Availability

Study data is available on demand by contact with the corresponding author.
